# Modification of Porro’s Operation

**Published:** 1886-09

**Authors:** John Bartlett

**Affiliations:** Fellow of the Chicago Gynæcological Society; 482 North Clark Street


### A PROPOSED MODIFICATION OF PORRO'S OPERATION. *By* JOHN BARTLETT, M. D., *Fellow of the Chicago Gynæcological Society.*

[Read before the Chicago Gynæcological Society, June 18, 1886.]

MR. PRESIDENT:

I desire to present to the Society this evening a method of procedure in cæsarian section which possibly may in some cases be substituted for Porro's operation.

No operation in surgery has so interested the world as that of cæsarian section. By some, records of it are considered to be as old as written language. Not only has it interested surgeons; it has engaged the attention of legislators and ecclesiastics. Invested with the glamour of superstition and romance it has played a part in history. The progress of the operation may here be briefly outlined. Though so few early authentic records of cases have been handed down to us in the annals of surgery, it is yet certain that in the time of the first systematic writer on obstetrics, the operation was frequently put in practice—Mauriceau says, speaking of his own times, "it is done by ignorant persons every day in the country," and so great and frequent were the abuses of the operation after his day that a number of the most distinguished surgeons and obstetricians of Europe condemned and opposed it.

Notwithstanding the very great interest with which the cæsarian section had been for so long regarded, the first suggestion as to its improvement seems to have been made by Michaelis, of Marburg, in 1809. He is quoted by Maygrier as recommending that extirpation of the uterus be combined with cæsarian section, to the end that a woman with a pelvis so narrow as to preclude natural birth could not again become pregnant. As cited by Harris, he said, "It is indeed a question whether the cæsarian section would be made less dangerous if with it were combined the extirpation of the uterus."

In 1828, Dr. James Blundell, of London, in his classical midwifery, wrote in his chapter on cæsarian section as follows: "In the hope of unlocking the abdomen for surgical operation, I have myself endeavored to prove that extensive divisions of the peritoneum are not in general followed by fatal results. * * * When I first turned my attention to the profession I used formerly to hear that, like wild fire, an inflammation, commencing in a spot of the peritoneum, might be expected to spread rapidly over its whole surface. That the risk of diffused peritonitis has been greatly exaggerated, I have endeavored to show in a small paper, and from the adverse opinions of my contemporaries on this point I confidently appeal to posterity. In some future age, when our hearts and their petty passions are quiet in the dust, this opinion, whether right or wrong, of great importance to our race in all future ages, will probably be decided by accumulated experience—may I not add, in the affirmative? to this dijudication I think it better to commit it. * * * In speculative moments I have sometimes felt inclined to persuade myself that the dangers of the cæsarian operation might, perhaps, be considerably diminished by the total removal of the uterus. If the cæsarian section be performed on a rabbit in the ordinary way, unless I am much mistaken, it will be found that the animal generally perishes in consequence. But in four rabbits recently delivered, I made an opening above the symphysis pubis, and raising the womb from the abdomen, I elevated them above the aperture. * * * Having applied my ligatures about the vagina I completely removed the wombs. * * * I (then) drew forward the ligatures till I brought the raw surface, left by the removal of the wombs, in contact with the abdominal incision internally, * * * trusting that this might promptly contract adhesion with the inner surface of the abdomen. Beyond my hopes the operation succeeded; of the four rabbits, three recovered, the fourth dying in consequence of the ligatures slipping from their place. In performing cæsarian delivery on the human body, perhaps this method of operating may hereafter prove an eminent and valuable improvement."

Lately the interest in the cæsarian section has greatly increased. It has been stimulated by the successes in other lines of abdominal surgery. Surgeons, the world over, have been impressed with the possibility of decreasing the fatality of this operation by applying to it the principles which have grown out of Listerism. A hopeful confidence so aroused has led to more frequent and earlier resort to the operation, and has placed it more and more in competition with craniotomy. Interest in it has been greatly enhanced of recent years also by the adoption by surgeons of the method of operating practiced by Dr. Porro. Dr. Porro some years since, struck by the fatality of cæsarian section, and reflecting upon the cause thereof, conceived the operation which has since borne his name. Many of the unfavorable results of the old operation are to be ascribed in the main to the impossibility of so closing the wound in the uterus, that closure may be maintained. The incision in the womb, the relations of whose walls are constantly changing, could not be stably united by a suture, which would prove insufficient after a few hours, because of changes in the relations between the knotted loops of ligature and the ever altering tissues which they embraced. Dr. Porro cut the Gordian knot of this problem by the removal of the entire body of the uterus,— a procedure which also eliminates the dangers of hæmorrhage from the uterine incision as well as those incident to the open wound presented by the placental site.

In the Porro operation the danger of shock from the ablation of the uterus and of complications arising from the management of the abdominal wound, especially those incident to traction on the pedicle and the presence of a drainage tube, when this is used, are superadded to those of the cæsarian section.

The essential features of Porro's operation are the seizure of the emptied womb by a wire clamp at such point as will admit of retaining the amputated stump within the abdominal wound. The wire ligature is tightened from time to time if bleeding threatens, and is allowed to remain until the projecting uterine tissues on its distal side have lost vitality.

In the hands of some special operators the results of the method have proved most promising; thus in the maternity of Milan, of fourteen women operated upon, eleven were saved.

According to Dr. R. P. Harris, in the Encyclopedia of Surgery, "the Porro operation has entirely superseded the old method in most of the lying-in hospitals of Europe, and has reversed their tables of mortality completely. Where for many years every patient died, now at least 50 per cent, and in some hospitals a larger proportion, recover. It is true, antiseptic measures, preparative treatment and early operating have had much to do in effecting this, but there is also a very decided diminution of the death rate to the operation itself. It has failed in Great Britain in nine out of ten cases, but this is due to the subject more than to the operation."

The latest statistics of the Porro operation (Harris) indicate in 151 operations 67 successes, giving 44.3 per cent of recoveries.

More recently, results of the Porro operation, as contrasted with the old method, especially when it was considered that every Porro operation had the advantage of the new modes of wound dressing, have been disappointing.

In the old operation the wound in the uterus under the new order of dressing, instead of being left to nature, has been in more recent cases closed with wire sutures, reinforced by superficial stitches, both so adjusted as to turn in the cut edges of peritoneum and retain the serous surface of one edge apposed to the corresponding surface of the opposite edge in a manner designated as "welting." Some autopsies have shown that this suture, at least in some instances, remained tight, although it has also been noted that the edges of the uterine wound corresponding to the uterine cavity are so drawn upon by the muscular fibre as to suffer eversion and thus present raw surfaces to the action of the uterine contents.

The statistics of more recent cæsarian operations (1874) in 1605 operations give a percentage of recoveries of 54.

In 1884 Prof. Mueller, of Bern, at the International Medical Congress, declared that the question as to which operation offered the better results, the old or the new cæsarian section, could not then be determined, inasmuch as the old method had not as yet been sufficiently tried under the new system of wound management.

Charpentier in his Midwifery says: "The tendency at present is to return to the old cæsarian operation."

The substitute for Porro's operation which I have to propose is as follows: The operation proceeds as in cæsarian section till the child is removed, the actual cautery being used in opening into the womb. Then, instead of dragging the womb out of the abdomen through the abdominal incision it is dragged out of that cavity through the vagina. The operator passes a Well's clamp somewhat modified in its prehensile surfaces and properly curved in coincidence with the parturient canal to the fundus of the uterus and there secures a firm grasp into the uterine tissues. By traction upon these forceps, and pressure, and suitable manipulation from above, the fundus of the uterus is depressed into the body of the organ, and drawn through the cervix into the vagina to produce complete inversion. The clamping wire is immediately adjusted, and excision of the uterus and appendages effected at a suitable distance from the vaginal junction. The abdominal wound is closed, and attention is given to the stump in reference to hæmorrhage as in Porro's operation. In lieu of the clamping forceps in some cases it would answer better, doubtless, to pass a loop of copper wire through the walls of the uterus to be caught upon a suitable instrument, as a rod possessing the flexibility of block tin or solder, passed *per vias naturales* to receive it. The advantages of this operation over Porro's method which suggest themselves are: *First*, that the abdominal cavity is thoroughly closed; the abdominal incision, not being embarrassed by the presence of the large pedicle, is as perfectly and as quickly closed as in any other laparotomy. By the process of inversion the pedicle is placed outside of the abdominal cavity, while what may be termed the uterine inlet made into the peritoneal sac is closed by the clamping wire opposing serous surface to serous surface, thus offering the best prospect for speedy and certain agglutination and closure. *Second*, the relation of the parts in the suggested procedure are much more natural, and much less strained than in the *status* in which Porro's method leaves them. *Third*, in the event of drainage becoming necessary in the course of treatment, the effecting of an opening for a tube in the plan proposed can be accomplished very much more easily and safely than in Porro's plan, and the tube being introduced, its situation and direction would be the best possible for thorough cleansing of the cavity to be washed.

Serious objections, at first thought, will occur to the mind of every gynæcologist. These will be here stated and subsequently met, as well as may be, by considerations that may be urged in answer to them.

*First:*—Of all the accidents *post partum* none is generally accredited with so violent a shock to the patient as the very condition which is here made a main feature in a method proposed as conservative. In the old, and in Porro's operation it almost always happens that, either with or without the partial or complete separation of the placenta, the uterus contracts. With such a condition of the uterine walls inversion would prove difficult and sometimes probably impracticable. Hunter said a contracted uterus was as difficult to invert as a jack-boot. When to these difficulties incident to the first step of the operation are added the shock from clamping and incising the uterus, it would seem that the dangers incident to the method proposed might exceed those of the Porro operation.

*Second:*—In Porro's operation, as in the old cæsarian section, danger begins from hæmorrhage at the moment of incising the uterus, and in the method proposed this danger would be so much the greater as the time elapsing between the two events, incision and snaring of the pedicle, is longer. In the established operations in at least one-sixth of the cases the placenta has been encountered directly in the line of incision. In such instances the bleeding from the double wounds, uterine and placental, would, in an especial manner, embarrass the operator and endanger the patient.

*Third:*—It must be remembered that in the great majority of cases in which the operations under consideration are undertaken there exist contractions of the pelvis, which may seriously interfere with the main step of the operation, inversion of the uterus.

*Fourth:*—Apart from these more serious objections it may be urged against the plan by inversion that dilatation of the os uteri, a *sine qua non* of the method proposed, does not always exist at the time of operation, and that it may not always, or even often, be practicable safely to effect it.

These objections will now be considered seriatim. As to the first, regarding the shock to the system so often reported in association with inversions, it may be stated that associated with inversion also is very generally hæmorrhage, and that to this all-powerful cause of depression may be ascribed much of the shock noticed in cases of inversion. While it must be admitted that in some instances inversion alone, entirely unassociated with bleeding, seems to have produced great shock, and even death, it may yet have happened that in some of these cases, other injuries, as laceration of the uterus, accompanying the inversion may have been partly responsible for the profound impression observed, and one is the more justified in assuming that this objection may be over estimated, from the fact that in a number of cases carefully observed and reported, inversion has produced no shock whatever, and has in fact been accomplished without the knowledge of either the patient or obstetrician.

Blundell says "it sometimes happens that the patient scarcely sustains a single symptom of serious inconvenience (especially if reduction be speedily effected). It will sometimes happen that not a single pressing symptom shall occur."

Dailliez thinks that it is perhaps less dangerous than is generally supposed. He says "the character of the prognosis depends less on the accidents inherent to introversion than on the circumstances which precede, accompany or follow it. If the contrary opinion prevail it is because the accidents essentially dependent on inversion have not been sufficiently distinguished from those which result from its complications and efforts made to remedy it. Even the hæmorrhage, all other things being equal, is less copious and dangerous than that which sometimes follows delivery in inertia of the uterus without inversion."

Dugé says " death may have occurred from violence and not from the inversion *per se*."

Dr. Crosse, of Norwich, states that of 109 *fatal* cases of inversion 72 (only) perished within a few hours (66 per cent). Dr. Lee reports 52 cases; in 37 of these the uterus was restored in 24 hours, and of these only 5 died.

By reference to veterinary surgery cases may be adduced to show not only that uterine inversion among animals is not *per se* especially dangerous, but that inversion, complicated with accidents in themselves accounted most dangerous, is not necessarily fatal. In such cases reposition alone, unaccompanied with any care for existing uterine lacerations may be followed by perfect and speedy recovery.

A mare was delivered of a colt of about four months naturally.* A few hours afterward the veterinary found the uterus inverted and presenting a laceration of about two inches at its fundus. The hostler having seen the mare making violent efforts to extrude a mass of flesh like an after-birth, pulled upon the body, and in that way the tear had occurred. Reduction was accomplished with difficulty. The animal was fully convalescent on the eighth day, and completely recovered on the fifteenth.

M. Guillaumin was called to a cow which had just calved. The inverted uterus soiled with blood and debris of the placenta lay on the litter. A mass of intestines about a yard long had escaped through a laceration in the vicinity of the neck. The intestine was returned, and without attempting to approximate the edges of the laceration the womb was replaced. At the end of eighteen days the animal had resumed her usual regimen.

The following case was reported by M. Gellé, professor of veterinary surgery at Toulouse. A heifer, after a very laborious parturition, had an inversion of the womb. Reduction was effected two hours afterward. The uterine tissue, red and friable, did not withstand the pressure of the hand; perforation, through which the arm passed, resulted. Notwithstanding this accident reduction was accomplished; one hand

---

* From J. Rainard.

meanwhile preventing the escape of the intestines. The cow convalesced on the third day, and cure was complete on the tenth.

M. Eleout was called to a cow whose uterus was prolapsed and inverted. The left horn was reduced without difficulty; the right was also on the point of being reduced when the cow, by a sudden movement, thrust the horn of the uterus against the hand of the operator so that it pierced the organ and sank through it half way up the arm; nevertheless, the operator replaced the womb, retaining it in position by inserting a bottle as a pessary. On the fifth day the bottle was removed. On the fifteenth day the cow was in good health.

As to the objection regarding the difficulty of inverting the uterus after contraction, it must be admitted that contraction of the uterus into a firm body would certainly render more difficult the inversion. The facility with which the flaccid uterus may fall into itself like tripe, or a wet bladder or the finger of a glove, certainly contrasts strongly with the difficulties encountered by experts in restoring the inverted uterus, even as early as four hours after labor. In the absence of any experience in the matter of purposely inverting the uterus, it will be necessary in support of the practicability of this feature of the proposed operation to draw upon experience derived from practice in midwifery. A variety of facts may be brought to bear to show the likelihood of success in efforts at inversion which may be in a measure classified thus: Direct facts as to the ease with which it has been accomplished directly after labor;—facts showing the readiness with which from trifling causes inversion may be induced within a few weeks after labor;—facts seeming to show that it may even occur in the virgin uterus, and apparently from minor causes. Replacement of the uterus after inversion, whether that organ be lax, moderately condensed, or in a state of complete involution, is an act so nearly akin to that of inversion that any facts tending to indicate the facility with which an inverted uterus may be restored to position have a bearing upon the question of the practicability of inverting the uterine tissue. Hence in the category of available facts for our present purpose belong those showing facility, or possibility of reduction of the inverted womb at any stage or condition of inversion. Referring to inversion, Dr. Barnes says: "It may be supposed that the uterine ligaments would offer resistance to obviate spontaneous inversion, and the inference from this assumption would be that inversion must be the consequence of direct violence. But in reality this resistance is easily overcome. During gestation the ligaments become elongated, so as to be easily drawn into the hollow or cup formed by the inverting body of the uterus. The moorings of the fundus are too slack to keep this part up. The connections of the cervix to its ligaments, to the bladder and vagina, hinder the inversion of this part for a time." In this connection the words of Hunter may be cited: "The flaccid uterus is as easily inverted as the finger of a glove." By one writer the feel of it is given as that of a wet bladder, and another author assures us that nothing but the sustaining power of a temporarily devised stem prop would in one case prevent the relaxed fundus from falling down into the os after reduction. Several centuries ago when the practice of the obstetric art was almost exclusively in the hands of midwives, inversion of the uterus was common, and the accident is almost invariably referred by authors to the practice of pulling upon the cord. A practitioner informed Dr. Gooch that he once inverted the uterus by merely putting the cord on the stretch. A number of cases are reported where a short or shortened funis has produced inversion, and others where the weight of the child, when the woman has been delivered standing, has caused the accident. Barnes says "manual expression produced one case that he saw, and other similar instances are reported. "It has been produced by strong pressure on the abdomen." According to Boivin and Dugé, inversion has been produced by instruments employed for the removal of polypi. Baudeloque reports a case which occurred six days after labor; and Byford one which took place five weeks after delivery, from apparently slight causes; and cases are reported by other authors of inversion of the uterus in virgins. Instances are related in which inversion occurred and replacement was effected immediately after labor without suspicion on the part of the patient or attendants that anything unusual had occurred. Baudeloque reduced a recent case in half a minute. In another case in which the uterus had been inverted two hours, he replaced it in two minutes.

Radford reduced an inversion in fifteen minutes, although several days had elapsed since its displacement.

Mr. Charles Cowan reports a case caused 80 hours after labor by the patient's rising suddenly out of bed. Prof. J. Y. Simpson reduced the inversion five or six days after labor, "in a short time with little difficulty." And, instances wherein replacement has been effected as late as one week after labor are on record. Baudeloque recorded one sixteen days after parturition.

The caution given by every teacher of midwifery against the employment of anything like forcible traction on the cord in efforts to deliver the after-birth, is in itself a strong intimation that the uterus may be readily inverted *post partum.*

Restorations of the inverted uterus years after the accident are now of frequent occurrence. In these restorative efforts the operators, apart from the great disadvantage which chronic continuance of the inversion, and the changes incident to that state presented, labored under the very serious disadvantage of most imperfect counter-pressure, and but little or no power of cervical dilatation. The achievements of gynæcologists in reinverting uteri years after their inversion in the face of these difficulties, furnish a strong presumption that an operator would not be foiled in his efforts to invert the uterus as a step in the procedure recommended, especially as in the circumstances here suggested, the uterus is exposed to manipulations *a tergo*, as well as *a fronte*, so that every facility is presented to the surgeon in his attempts at inversion.

It is to be considered also that many of the cases on record where difficulties in replacing an inverted uterus were extreme, occurred before the discovery of anæsthesia. From statements made rather recently it would appear that the obstetrician of today has at hand means of overcoming the spastic contraction of the uterus *in toto.* For very many years the student of obstetrics has been taught to apply a preparation of belladonna to the cervix in case of rigidity during labor; and, notwithstanding the general incredulity as to the efficacy of the means, it still holds its place with some writers as an efficient agent in these conditions. About 10 years ago (quoting from the half-yearly *Compendium of Medical Sciences*) Dr. E. Fraenkel was led, by the experiments of Breslau on the effect of the subcutaneous injection of atropine on uterine contraction, to turn his attention to this drug. Having employed it in combination with morphia in many cases, he has come to the following conclusion: In cases of spastic contraction of the uterus, either in the second or third stage of labor, whether the whole or only the part of the uterus is involved, the use of the combined subcutaneous injection of atropine and morphia, followed by the administration of chloroform, is the safest, surest and qu ckest method for overcoming uterine spasm, and rendering operative interference possible.

Dr. Fraenkel recommends these agents, among other conditions, to abate "violent contraction of the uterus met with during the third stage, due to premature attempts to remove the placenta." This combination recommended by Dr. Fraenkel, injected into the cervix uteri at the proper moment before the operation, might be relied upon to antagonize any excess of contraction of the uterus which experience might show to interfere with the efforts of the operator to invert the uterus.* In regard to the objections having reference to hæmorrhage from the uterine incision it will be observed that in the plan proposed the incision through the uterine walls is made with the cautery. While it is probable that the protecting power

* Fraenkel states that the use of atropia in spastic uterine contractions "got into disrepute through a case published by Spiegelberg, in which the subcutaneous injection of one-fortieth of a grain was followed by dangerous atony of the uterus," and he recommends that the dose do not exceed one-sixty-fourth of a grain. The fact that the one author obtained an excessive action of the medicine, and that the other limits accurately the dose, suggests the power of the agent to bring about the condition of the uterine tissues desired in efforts at inversion. It will be noted that the circumstances under which it is here proposed to use atropia render the degree of atony induced of less consequence than in cases of labor. For, in the case here supposed, the ligature is to take the place of the contraction of uterine fibers in controlling hæmorrhage.

of this agent would guarantee the arrest of the bleeding from the uterine wound for a time under conditions of rest, it must be admitted that, in subjecting these seared edges to the changes of relation incident to the process of inversion, there would be danger of re-opening the vessels and loss of blood. In such a case the assistant managing the thermo-cautery would follow the edges of the wound with the purpose of re-touching bleeding points when practicable. That the actual cautery may arrest the hæmorrhage from the uterine wound, even under circumstances of change in its size, etc., the following fact proves.

Dr. R. W. Felkin witnessed a successful case of cæsarian section by a native operator at Kaeura, Central Africa. From Dr. Breitmann's account it will be seen that the bleeding was staunched by the actual cautery. After the extraction of the child the operator grasped the uterus and compressed it with both hands with all his might. "What bleeding still continued was stopped by the actual cautery, the operator still compressing the uterus until it was firmly contracted. In regard to hæmorrhage in eighty-eight cases cited by Playfair, the particulars of which were carefully noted, severe hæmorrhage occurred in 14, six of which terminated successfully, and in four only could the fatal result be ascribed to the loss of blood. In one of these the source of hæmorrhage is not mentioned, in another it came from a wound in the abdominal wall, and in the other two from the uterine incision being made directly over the placenta. In neither of the two latter was the loss of blood immediately fatal; for it was checked by uterine contraction and only recurred after many hours had elapsed."

Says Baudeloque, and others endorse his statement, "There is little blood discharged from the wound in the uterus when it has been made in the middle of the anterior part; except the placenta be attached there, and even then the hæmorrhage does not last long, if the uterus contract firmly."

Dr. James Edmunds says, in reporting a successful case of cæsarian section in which the placenta lay in the line of incision, "I now cut into the uterus; on deepening the incision blood gushed rapidly out: I instantaneously plunged the knife completely through the uterus and placenta and thrust two fingers through the wound and removed the placenta. "After careful examination, we estimated the entire loss of blood at about fifteen ounces."

James Whitehead reports in his case of cæsarian operation; "The cut into the uterus was about seven inches long. A small incision was first made with the scalpel and continued with the probe pointed bistoury. The loss of blood amounted to about eight or ten ounces; it issued principally from the placenta while being torn through. There was scarcely an ounce of blood from the divided edges of the uterine parietes."

Robert P. Harris says "hæmorrhage is rarely the cause of death."

As to the objection regarding the occasional wounding of the placenta, it holds, in a much less measure, however, as to Porro's operation as well. In such cases the cautery knife would have to be mainly relied upon as in other instances, where the uterine wound alone is concerned. The placental bleeding would be in some measure controlled by it, and, as aids, the ovariotomy forceps of Sydney Jones as recommended by Fancourt Barnes might be depended upon to assist in restraining the hæmorrhage while the child was being delivered. After the removal of the placenta, bleeding from its site, if profuse, should be combated by the application of the solution of the perchloride of iron, or by a cautery plate, as experience should show to be preferable. In regard to the third objection having reference to the narrowing of the pelvis and the difficulties in the way of the suggested procedure thereby presented, it may be stated that while narrowing of the pelvis would always prove more or less of a hindrance, yet it must be borne in mind that in the majority of cases of deformed pelvis, however much any given diameter may be shortened, there yet remain spaces to one or the other side of the narrowing line through which the womb might be made to pass by the *vires a fronte et a tergo.* Generally in the process of inversion, as the uterus would be drawn through the superior strait, four thicknesses of the organ would be presented at the conjugate; and in cases of unusual narrowing, difficulty might be experienced in this manœuvre. In extreme contraction of the pelvis, dexterity and ingenuity on the part of the operator might enable him to cause the organ to pass in the process of inversion a very narrow space, possibly no wider than twice the thickness of the uterine parietes. Thus by making the incision, where practicable, near the fundus the fold formed by one lip of the wound and its apposed surface of uterine wall might be made to pass; to be followed by a similar fold of the corresponding edge of the incision.

Stein and Weigand recommend that after the operation of cæsarian section if the uterus does not contract so as to sink into the pelvis, it shall be seized by the whole hand, as in taxis for hernia, and be pressed down into the pelvis. In a narrow brim, this procedure, they think, insures that the uterus once pressed into the pelvic cavity cannot rise out of it again. Spitzbarth makes a similar suggestion. These recommendations of practical men suggest the feasibility of inverting the uterus by adroit manipulation even in cases of marked contraction. It may as well be stated, however, that the plan of operation here proposed has its limits of practicability as compared to the Porro operation, cases of extreme pelvic obstruction as well as those involving such changes in the parenchema of the uterus as would render inversion dangerous, if not impracticable, would, of course, not fall in the category of those to which the method here suggested might be applicable.

In regard to the fourth objection as to the hindrance presented by a non-dilated os uteri; it may be said that according to the majority of authorities, the most favorable time for performing cæsarian section is after labor has set in, and should interference be delayed till the os uteri were softened and ripe for dilation in the greater number of cases the delay would not prove injurious to the mother or child.

With the present means of dilating the cervix during labor, it is to be presumed that while an imperfectly dilated os would no infrequently prove a hindrance it would not often be an obstacle in the way of the proposed operation. Barnes may here be cited; speaking of the induction of labor, he says: "By the aid of the water bag the cervix may be expanded sufficiently to admit of delivery within an hour. * * * In cases of placenta prævia we have effected adequate dilatation in half an hour." Dr. Lusk says the rigid cervix can be forcibly dilated to almost any extent by hydrostatic pressure*; and other writers refer to this agent as a means which enables them to set almost the hour of delivery when necessity for forced labor exists.

Dr. Thomas, in his operation for very obstinate cases of inversion, found no difficulty, by means of his peculiar dilator, in dilating the cervix prior to reposition. It may be presumed, therefore, that in cases demanding cæsarian section, if necessary,

* Dr. Lusk gives a caution against the abuse of this power.

dilatation of the os uteri sufficient for inversion could generally be safely effected. As adjuncts to the more decided measures, the hot water douche, and the local hypodermatic use of atropia and morphia might be relied upon.

It may be well here to refer to some details of the operation. It is obvious that in the adjustment of the ligature it would be desirable to make the inversion complete. Experience alone could determine the point of election for placing the constricting wire. All that portion of the neck of the uterus lying after labor below the vaginal attachment would be entirely out of the way of the operation, and it would be likely that the line of junction of this collar of flesh would serve as an index of the proper point for ligation. It will be noted that in the case reported by Boyer, after a number of days the ligature was no longer within reach. It will be borne in mind, as a useful hint, that in the case presently to be cited where ablation of the inverted uterus was made in the bitch, the stump of the organ sprang suddenly inward. These facts are suggestive. They point out the fact that the ligation and abscission is in a manner a liberation of the tissues forcibly drawn out of position by the act of inversion, a liberation greatly tending not only to diminish shock and to relieve discomfort, but also to lessen the liability to any inflammation which extreme dragging upon the pedicle might incite or foster. Attention is called to the fact that where reference has been made by observers to the character of the stump after the cure, it is often spoken of as presenting an appearance very similar to that of the os in the natural state. Where the line of ablation is somewhat above (anatomically) the infra vaginal cervix it is drawn so far upward as to free the cervical collar, and the fibres of this ring reasserting themselves close below the stump and "reconstruct" the os tincæ.

It may be inquired, What would be the relation of the ovaries to the proposed line of ligature in an inverted womb? Several writers refer to the ovaries as resting on the edge of the inverted uterus as if about to fall into the cavity. A specimen from which this statement has been deduced forms the original of one of the standard cuts representing that condition. It is a case of partial, not of complete, inversion. Some authors, as Boivin and Dugé, state that the ovaries are not within the cavity of the uterus. Other writers, as Levret, report cases in which the ovaria were found within the inverted cavity. Schultze states that they are there found, and the cut that accompanies his text so shows these organs. In a number of instances, recent and old, the amputated uterus has been found to contain one or both ovaries. In many cases of chronic inversion the appendages have not been found within the cavity of inversion. A study of the relation of the ovaries after complete inversion of the uterus will lead to an indorsement of the statements of Winckel and Schroeder as correct. Winckel writes, "In puerperal inversion, as a rule, the tubes and ovaries fall into the cavity (of inversion)."

Says Schroeder, "In recent puerperal inversion all of the appendages are in the uterine funnel."

In the records of medicine are not wanting quite a number of cases the history of which teaches that the plan of operation here proposed may not be fatal. Some of these are here appended.

* Obs. 170. Perrachi, in 1837, reports that a midwife, in a case of inversion of the uterus occurring during labor, so

---

* NOTE. The cases here numbered have been translated from the work of Denucé on Uterine Inversion.

dragged upon the tumor as to pull away the womb. Nevertheless recovery resulted.

Obs. 171. Kuhlbrand, in 1839, refers to a case of inversion in which part of the tumor was pulled away. Recovery followed.

Thomas cites a case, 1842, in which "one child being born, the midwife felt the breech of another, as she supposed. Around it she passed a handkerchief, pulled with all her force, and dragged away uterus and annexæ. The patient recovered."

Obs. 173. A bone-setter, as reported by Breslau, in 1850, violently dragged away the inverted uterus and its appendages. A cure took place in seventeen days. There remained, however, at the upper portion of the vagina, an ileo, or recto-vaginal fistula. Finally, after a time, fecal matters resumed their usual course.

Obs. 175. X——— in 1851. A midwife tore away the uterus and one of the ovaries by violent traction on the placenta. Nevertheless recovery resulted.

Obs. 174. Oldham in 1850 reports that a midwife pulled upon the uterus, inverted it and tore it partly off. She completed the ablation with a bistoury. The woman recovered.

Obs. 172. Rossi reports a similar case. A midwife, by immoderate tractions, detached a great part of the uterus, and finished the operation with a knife. Restoration was very rapid.

Obs. 167, 168. Velpau, in 1836, cites two cases, one related by Viradel, the other by Caille, in which midwives excised inverted uteri. Both cases ended in recovery.

Obs. 164. Bernhard, in 1802, on the authority of Breslau, relates the experience of a midwife, who, on the occasion of an inversion occurring at the time of labor, cut off the uterus with a razor. The hæmorrhage, which was violent, was arrested by ice. The patient recovered with a rectocele.

Obs. 34. Wrisberg, in 1782, reports the fact that a midwife, in a case of inversion produced by violent tractions on the placenta, had the temerity to resect the womb with a knife. Torrents of blood flowed; hæmorrhage was arrested by syncope. Wrisberg was called two days afterward. He recognized an opening two inches in diameter between the vagina and the abdominal cavity almost closed, however, by the bladder filled with urine. The condition of the woman improved from day to day, and at the end of three months she was able to call upon Wrisberg.

Obs. 163. Osiander, professor at Gottingen, about the year 1800 reports that a midwife, having drawn the womb with the placenta out of the vagina, cut it off at the level of the latter without the ablation proving fatal.

Slevogt, a surgeon of Jena, removed the uterus and fallopian tubes, under the impression that he was merely excising an excrescence; the woman perfectly recovered.

Vieussens removed the uterus for inversion successfully, only a small portion of the womb remaining.

Obs. 110. Clemencen mentions the fact of a complete inversion of the uterus, followed by gangrene, in which recovery took place.

Murray reports a case, says Barnes, where the uterus was found inverted twenty-four hours after spontaneous expulsion of the placenta. It was released, and again found inverted on the following day. On the thirteenth day it sloughed off without hæmorrhage. The patient recovered.

Rousset, 1581, Primrose, and Radford have given cases in which the inverted uterus appears to have sloughed off without compromising the patient's life.

Obs. 32, Faivre, 1767, surgeon of Vesoul, reports as follows: A midwife delivered a young woman, usin; great violence in delivering the placenta. Finding the uterus inverted she pulled upon it with all her force with no other effect than to increase its volume. A day or two after, the uterus having fallen into gangrene, Faivre applied a ligature. Twenty-seven days thereafter, the patient meanwhile suffering greatly from fever, convulsions, etc., the uterus fell off. After a month of fœtid suppuration the wound cicatrized. The woman recovered her strength with astonishing rapidity.

Obs. 169. Cooke, in 1835, relates the following case: A midwife dragged upon an inverted uterus, which she mistook for a second child, and partly detached it. Cooke was then called and applied a ligature above the mutilated portions of the uterus; these he retrenched. The uterus contained the right ovary and the fallopian tubes. The woman recovered, without accident, other than the temporary induration of a breast.

Obs. 201. In Bradley's case, 1874, complete inversion was produced by the midwife's pulling on the cord. Dr. Bradley was called 15 days afterward. The uterus, much inflamed, presented a spot of gangrene as large as a dollar; the pulse was 120, and the countenance hippocratic. Having tied the vaginal arteries he amputated the uterus. In one month the woman was walking about the room. Her recovery was complete.

Obs. 235. Hunter, 1799, on the eighth day after labor applied a ligature to an inverted womb, and six hours later excised the organ, no pain, no bad symptom arose after the operation. The patient left the bed on the 15th day and was well at the end of a month.

Obs. 165. Rottger, in 1820, citing Breslau, reports a case of inversion due to a polypus. A bone-setter excised the tumor piecemeal. This rash operation led to a violent hæmorrhage. Rottger, called in time, succeeded in ligating the uterus and excising the part below the ligature. The woman recovered.

Obs. 166. Weber mentions an inversion due to a polypus. A midwife having pulled away the tumor, gangrene occurred. Weber placed a ligature on the vagina and amputated beyond it on the fifth day. The patient recovered.

Obs. 31. Anselin, surgeon of Amiens, 1764, reports a case in which an inversion of the uterus recurred after primary reduction on the 15th day, forming a tumor as large as an ostrich egg between the thighs. Anselin applied a ligature and seven days afterward amputated the organ. In seven weeks the woman was entirely recovered.

Dr. Cleghorn, of Glasgow, 1810, reports the following case:

"At the end of half an hour after a natural but laborious labor the placenta had not separated. The midwife proceeded to remove it; in doing which she employed so much force that she dragged out the after-birth and uterus together. * * * The patient fainted away; but on her recovery she found there had been a profuse flooding, and that there was a large tumor externally. No attempt was made to return it, and she therefore remained in this situation for three weeks, when with the greatest difficulty she walked to Welshpool, a distance of nearly three miles, where she received medical attendance. The exertion of walking brought on a very violent inflammation of the parts, which during the first fortnight was followed by a great sloughing of the coats of the uterus, attended by a very fœtid ichorous discharge. A fortnight after this time, and about five weeks subsequently to the accident, it was found that reduction of the tumor was become impracticable, and that extirpation was the only chance left for putting an end to the poor woman's sufferings. The whole of the uterus, and at least an inch and a half of the vagina, were excised. The patient in about a fortnight began to improve in her flesh, and in six weeks after the operation she was perfectly recovered."

Several histories of *post partum* accidents occurring in animals which cannot fail to bear upon the questions involved in this paper are here appended. They are extracted from an admirable work published at Lyons in 1850, entitled "*Traité complet de la paturition des principales Femelles Domestiques, par J. Rainard.*"

M. F. Huillet reported the following: A cow calved without accident; two hours afterward inversion occurred. The veterinary proceeded to replace the organ, and when the reduction was partly effected the parts were thrust so rudely outward by violent expulsive efforts that the hand perforated the fundus, and out of the accidental opening there escaped a considerable mass of small intestines. This was the sixth case of the kind which had fallen under the observation of M. F. Huillet and as yet he had never succeeded in remedying the accident by suture. He therefore resolved upon ablation of the viscus which had been perforated. After having carefully returned the intestines through the laceration, which was not less than six or seven inches in extent, he applied a double ligature, reinforced by a circular one, and performed ablation about an inch and a half beyond the ruptured portion. After one month the cow could do her usual work.

A cow experienced after parturition inversion of the uterus. Reduction was effected, four or five hours after the inversion recurred; a second restitution followed, and a second recurrence of the inversion. The uterus, now in a state of congestion, was incapable of replacement because of its increased size. Lotions were applied for three days, when a dog fell upon the mass of flesh with his teeth, tore the mucous membrane in several places and caused a fairly abundant hæmorrhage. Inflammation followed, and on the tenth day all hope of returning the tumor being lost, ablation was decided upon after the following plan: The animal being on her feet and the uterus sustained horizontally by two assistants the veterinary made on the superior wall of the organ an incision about six inches long, traversing the thickness of both walls. Between the margins of this opening he inserted a roll of linen six inches long and four inches in circumference, and by means of a tape two yards long, used as a double headed roller, he applied a series of turns very tightly about the pedicle of the tumor; that is to say about that portion of the uterus embraced by the structure of the neck. The ninth day all that portion of the uterus beyond the ligature was amputated. About this time the cow had regained her appetite, the fever had ceased, and a short time thereafter she was able to work.

In a case of inversion of the uterus in a bitch under the care of M. Cros, a ligature was placed as nearly as possible to the uterine neck. On the third day the strangulated tissue was amputated an inch beyond the ligature. There followed very little loss of blood. From the moment of incision the inverted stump of the uterus withdrew suddenly dragging the ligature. A serous flow appeared on the second day; on the fourth day the ligature came away.

Meanwhile the general health of the animal was not seriously deranged.

"There have been published a number of cases of extirpation of the uterus in the cow, the goat, the sow and the bitch followed by complete recovery. Generally the occasion has been rupture of the organ and escape of the intestines in consequence of the uterus being torn by dogs."

M. Chanel has seen the half of one of the cornua of the womb of a sow expelled from without the abdomen, cut off, together with the two fœtuses therein contained. The other fœtuses of the same cornus continuing to develop to the end of gestation.

Mr. President: In the course of my researches in preparing this paper,* I have looked expectingly for the presentation of the same proposition as I have here made from colaborers in the field of obstetric surgery. I

---

* After writing this article the writer found in the essay of Dr. Harris on the Porro operation in continental Europe, published in the American Journal of the Medical Sciences, in 1880, the following sentences:

"Several other plans [of treating the cervix] have been proposed. * * * (2) to invert the uterus after its evacuation and constrict and remove it by the vagina. This plan tends to complicate the case and increase its dangers, etc."

Had the writer been aware that the suggestion which forms the basis of the foregoing paper had been previously published he would not have prepared it. Inasmuch, however, as the merits of the method proposed are in no wise affected by its having been previously suggested, he has decided not to withhold the article from publication.

Dr. Harris, in objecting to the plan of leaving the pedicle in the vagina, says: "Suppuration within the vagina is not nearly so safe as it is external to the abdomen, where purulent absorption is much less likely to occur, and especially under the dressing of Lister."

There are reasons to believe that Dr. Harris has an exaggerated idea of the danger from this source, for in 63 cases of removal of the chronically inverted uterus by the form of ligature here recommended, there occurred a mortality of only 15 per cent, and if the cases be eliminated in which the patients died from shock, exhaustion, cancer, etc., the result shows a mortality of only 10 per cent. If it were practicable to eliminate the dangers incident to and inseparable from amputation of the uterus *per se*, the element have been rather surprised to have met no allusion to the method. The germ of the plan here proposed may however be found in the writings of that brilliant obstetrician to whom, more than any other, suggestions for improvement in the operation of cæsarian section are to be credited, James Blundell. In his article on laceration of the uterus occur these words: "Would extirpation of the uterus, *with or without inversion*, be of service in these cases? This question may be answered next century."

482 North Clark Street.

---

of mortality to be charged to the presence of the stump in the vagina would certainly be small. If deemed necessary, the pedicle within the vagina might be subjected to constant irrigation.

It has occurred to the writer that, so far as he is aware, in performing the cæsarian operations surgeons have not sufficiently availed themselves of their anatomical knowledge and surgical resources in the direction of controlling hæmorrhage from the uterus.

There would appear to be no reason why this hæmorrhage might not be controlled by two skilled assistants, one on either side of the patient.

The uterine artery, it is thought, could be controlled by pressure with the finger backward, outward and upward on the internal iliac artery. The ovarian artery might be controlled by the thumb pressing it backward against the body of the psoas muscle, about on a line with the bifurcation of the aorta.

Pressure upon the vessels should not be made until the uterine incision begun, nor relaxed till the constriction of the cervix has been effected.

Should this method of restraining hæmorrhage during the operation prove successful, the plan of bringing the cervix inverted into the vagina would be greatly simplified, for the uterus could be ablated at the point of election *in situ*, and then, by traction upon wire loops introduced near the incised edge of the cervix, it could be inverted and the clamping wire adjusted. The dragging of the body of the womb through the pelvis and os uteri could in this way be avoided.

